# Extreme hyperuricemia is a risk factor for infection-related deaths in incident dialysis patients: a multicenter prospective cohort study

**DOI:** 10.1080/0886022X.2020.1788582

**Published:** 2020-07-14

**Authors:** Hiroyuki Yoshida, Daijo Inaguma, Eri Koshi-Ito, Soshiro Ogata, Akimitsu Kitagawa, Kazuo Takahashi, Shigehisa Koide, Hiroki Hayashi, Midori Hasegawa, Yukio Yuzawa, Naotake Tsuboi

**Affiliations:** a Department of Nephrology, Fujita Health University School of Medicine, Toyoake, Japan; b Department of Nephrology, Nagoya University Graduate School of Medicine, Nagoya, Japan; c Faculty of Nursing, School of Health Sciences, Fujita Health University, Toyoake, Japan; d Department of Preventive Medicine & Epidemiology, National Cerebral and Cardiovascular Center, Osaka, Japan; e Department of Nephrology, Fujita Health University, Bantane Hospital, Nagoya, Japan

**Keywords:** Hyperuricemia, dialysis, infection, mortality, dialysis initiation, cohort

## Abstract

**Introduction:**

There are few studies on the association between serum uric acid (UA) level and mortality in incident dialysis patients. We aimed to clarify whether the serum UA level at dialysis initiation is associated with mortality during maintenance dialysis.

**Methods:**

We enrolled 1486 incident dialysis patients who participated in a previous multicenter prospective cohort study in Japan. We classified the patients into the following five groups according to their serum UA levels at dialysis initiation: G1 with a serum UA level <6 mg/dL; G2, 6.0–8.0 mg/dL; G3, 8.0–10.0 mg/dL; G4, 10.0–12.0 mg/dL; and G5, ≥12.0 mg/dL. We created three models (Model 1: adjusted for age and sex, Model 2: adjusted for Model 1 + 12 variables, and Model 3: stepwise regression adjusted for Model 2 + 13 variables) and performed a multivariate Cox proportional hazard regression analysis to examine the association between the serum UA level and outcomes, including infection-related mortality.

**Results:**

Hazard ratios (HRs) were calculated relative to the G2, because the all-cause mortality rate was the lowest in G2. For Models 1 and 2, the all-cause mortality rate was significantly higher in G5 than in G2 (HR: 1.63, 95% confidence interval [CI]: 1.14–2.33 and HR: 1.78, 95% CI: 1.19–2.68, respectively). For Models 1, 2, and 3, the infection-related mortality rate was significantly higher in G5 than in G2 (HR: 2.75, 95% CI: 1.37–5.54, HR: 3.09, 95% CI: 1.45–6.59, HR: 3.37, and 95% CI: 1.24–9.15, respectively).

**Conclusions:**

Extreme hyperuricemia (serum UA level ≥12.0 mg/dL) at dialysis initiation is a risk factor for infection-related deaths.

## Introduction

Uric acid (UA), a potent antioxidant, is the end-product of purine metabolism and plays a role in the elimination of nitrogenous compounds [1,2]. However, the function of UA in humans is not completely understood. Hyperuricemia directly or indirectly leads to various diseases, including gout, urolithiasis, chronic kidney disease (CKD), and cardiovascular (CV) disease [3–8]. Meanwhile, severe hypouricemia causes acute kidney injury induced by anaerobic exercises [9]. CKD is also a major cause of secondary hyperuricemia due to the decreased urinary UA excretion. The increase in the number of patients with CKD worldwide is concerning, because, often, CKD not only progresses to end-stage kidney disease (ESKD) but also leads to CV disease [10,11]. Various factors, including proteinuria, anemia, and hypertension, are associated with CKD progression, CV disease incidence, and all-cause mortality [12–14]. Regarding UA, some reports have revealed that hyperuricemia is related to a decline in the glomerular filtration rate (GFR), incidence of CV disease, and mortality [15–18]. Liu X et al. conducted a meta-analysis and indicated that UA-lowering agents might be effective in retarding the CKD progression [19].

The Kidney Disease Improving Global Outcomes (KDIGO) 2012 Clinical Practice Guideline for the Evaluation and Management of CKD indicates that evidence supporting the reduction of serum UA levels for the protection of kidney function is lacking [20]. In other words, it is still unclear whether the serum UA levels in pre-dialysis and dialysis patients are significantly associated with mortality. According to the results from several studies, it is possible that both hyperuricemia and hypouricemia are risk factors for mortality [21,22]. However, only a few previous reports have evaluated this relationship in dialysis patients [23–26]. Especially, the relationship between serum UA levels and infection-related mortality has not been reported in even if not dialysis patients though there are a report that suggested UA is one of biomarkers for evaluating severity of sepsis [27]. The serum UA level is extremely elevated in patients at the initiation of dialysis, despite receiving therapy for lowering the UA levels. This is because urinary UA excretion decreases because of a low GFR, and medications, such as allopurinol, are limited in their use because of their side effects, including skin eruption and leukocytopenia [28].

We previously conducted a multicenter prospective observational study to examine whether factors conditions, such as demographic variables and laboratory data at dialysis initiation, affected patients’ survival during maintenance dialysis from 2011 to 2016 in Japan [29]. Using the data obtained from this database, we aimed to clarify whether the serum UA levels at dialysis initiation are associated with mortality during the maintenance dialysis.

## Materials and methods

### Subjects

This investigation enrolled incident dialysis patients who participated in the Aichi Cohort Study of the Prognosis in Patients Newly Initiated into Dialysis (AICOPP), which was a multicenter prospective cohort study in Aichi Prefecture, Japan. A total of 1893 patients started dialysis for ESKD or acute kidney injury from 1 October 2011 to 30 September 2016, at 17 centers of the AICOPP group. Given that the purpose of this study was to examine the relationship between various factors at dialysis initiation and mortality during maintenance dialysis, we excluded 369 patients who died before discharge post-dialysis initiation. In addition, we excluded 34 patients whose serum UA levels were not measured at dialysis initiation. Thus, we finally included 1486 patients.

### Baseline characteristics and laboratory data

We set the baseline period as the time of dialysis initiation. The patients’ baseline characteristics, including demographic data, comorbidity, medical history, and medication, were obtained at the first hemodialysis session or first injection of peritoneal dialysate. We defined diabetes as a fasting blood glucose level of ≥126 mg/dL, random blood glucose level of ≥200 mg/dL, hemoglobin A1c level of ≥6.5%, or use of insulin or oral hypoglycemic agents. We defined a history of coronary artery disease (CAD) as a history of coronary artery intervention, heart bypass surgery, or ischemic changes on an electrocardiogram and stroke as a diagnosis confirmed by computed tomography or magnetic resonance imaging. As for the laboratory data, the blood sample at baseline was also obtained just before the first hemodialysis session or the first injection of the peritoneal dialysate. The following formulas were used to calculate the estimated GFR (eGFR) by sex: for men, eGFR (mL/min/1.73 m^2^) = 194 × age^−0.287^ × serum creatinine (mg/dL)^−1.094^, and for women, eGFR (mL/min/1.73 m^2^) = 194 × age^−0.287^ × serum creatinine (mg/dL)^−1.094^ × 0.739.

### Classification of patients by serum UA

We decided the cutoff points of the serum UA level as being 6, 8, 10, and 12 mg/dL, not defined by quartile, which we used to classify patients into the following five groups: G1 with a serum UA level <6 mg/dL; G2, 6.0–8.0 mg/dL; G3, 8.0–10.0 mg/dL; G4, 10.0–12.0 mg/dL; and G5, ≥12.0 mg/dL. Hyperuricemia is defined as a serum UA level of >6.0 mg/dL [30,31]. If we classified the groups by quartile, the cutoff points would be 7.2, 8.5, and 10.0 mg/dL. We considered that the hazard ratios (HRs) of the groups with normal and extremely high serum UA levels could not be accurately calculated. Moreover, when we considered the clinical situation, including treatment target, we thought that it was more useful to classify the patients using the aforementioned criteria.

### Outcomes

We evaluated the prognosis of patients every year after the start of the study until 30 September 2016, using medical records or mail for patients who were transferred to other institutions. The outcomes of this study included all-cause, infection-related, CV-related, and cancer-related deaths. We defined CV-related deaths as deaths caused by heart failure, acute coronary syndrome, fatal arrhythmia, and stroke.

### Statistical processing

SPSS statistics version 24 (SPSS, Chicago, IL) and the Easy R program [32] were used for statistical processing. We compared the baseline characteristics and laboratory data among the five groups using the analysis of variance (ANOVA) for continuous variables and Pearson’s chi-square test for nominal variables. All-cause and infection-related mortality were compared using the log-rank test for the Kaplan–Meier curves. We performed a univariate Cox proportional hazard regression analysis to detect factors contributing to all-cause and infection-related mortality. On the basis of the results and findings from previous studies on CKD and survival, we created the following models and performed a multivariate Cox proportional hazard regression analysis: Model 1: adjusted for age and sex; Model 2: adjusted for Model 1 + diabetes, CAD, stroke, vascular access, use of angiotensin-converting enzyme inhibitors (ACEs)/angiotensin receptor blockers (ARBs), allopurinol, loop diuretics, and thiazides, eGFR, and hemoglobin, serum albumin, and C-reactive protein (CRP) levels; and Model 3: stepwise regression adjusted for Model 2 + body mass index (BMI), primary kidney disease, history, or comorbidity of cancer, dialysis modality, use of β-blockers, statins, and erythropoiesis-stimulating agents (ESAs), and blood urea nitrogen (BUN), serum creatinine, serum adjusted calcium, serum phosphate, serum total cholesterol, and HCO_3_^-^ levels. We created Model 3 to compare its HR with that of Model 2 and to confirm reproducibility even after adjusting for all available variables by stepwise methods. In the stratified analyses, all-cause and infection-related mortality were compared using Cox proportional hazard models adjusted for the factors used in Model 2, because the all-cause and infection-related mortality rates were the lowest in G2. We selected use of loop diuretics, thiazides, and allopurinol as stratified factors that affected the serum UA level. *p* Values of <5% were considered statistically significant.

## Results

### Comparison of baseline characteristics and laboratory data

Table 1 shows the baseline characteristics and laboratory data of the five groups. Significant differences in age, duration of nephrology care, primary kidney disease, history of CAD, history and comorbidity of cancer, and use of ACEIs/ARBs, β blockers, allopurinol, thiazides, and ESAs were observed among the five groups. Regarding the primary kidney disease, we could not show the number of patients with gouty kidneys. However, we supposed that the number was very low, because the nationwide data from the Japanese Society for Dialysis Therapy shows that the incident dialysis cases caused by the gouty kidneys accounted for only 0.2% of all cases [33]. Among the patients with history and comorbidity of cancer, 92 patients had a cancer-bearing status. In addition, only 26 patients underwent chemotherapy, which might have increased their serum UA levels. However, none of the patient received chemotherapy just before dialysis initiation because of uremia. The mean serum UA level of the 26 patients who underwent chemotherapy was 9.1 mg/dL. Among these patients, only four patients had serum UA levels of ≥12.0 mg/dL. Hence, we included these patients in this study. Among the five groups, G1 has the highest proportion of older patients. The percentage of patients with a history of CAD was higher in G1 and G5 than in the other groups. The percentage of patients using allopurinol was higher in G1 and G2, whereas that of patients using thiazide was higher in G4 and G5 than in the other groups. The BUN, serum creatinine, and phosphate levels showed an increasing trend as the serum UA levels increased from G1 to G5.

**Table 1.** Comparison of baseline characteristics and laboratory data between the 5 groups by serum uric acid.

Variables	All N, 1486	G1 N, 136	G2 N, 430	G3 N, 543	G4 N, 245	G5 N, 132	*p* Trend
Age (years old)	67.5, 13.1	71.9, 10.4	67.7, 12.9	66.9, 13.1	66.5, 13.7	67.1, 14.1	.001
Female gender (%)	32.4	35.3	32.6	32.2	32.2	29.5	.906
BMI (kg/m^2^]	23.5, 4.4	22.9, 3.9	23.7, 4.2	23.6, 4.4	23.7, 4.7	22.9, 4.5	.143
Nephrology care (days)*	591 [168 − 1318]	532 [162 − 1361]	720 [260 − 1514]	590 [154 − 1358]	454 [84 − 1151]	328 [56 − 980]	.005
Primary kidney disease							
Diabetic nephropathy (%)	43.1	35.3	41.2	47.3	45.3	36.4	.004
Chronic glomerulonephritis (%)	14.9	17.6	16.0	14.2	16.7	7.6	
Hypertensive nephrosclerosis (%)	25.2	30.9	27.4	22.7	22.4	28.0	
Others (%)	16.8	16.2	15.4	15.8	15.6	28.0	
Medical history and comorbidities							
Diabetes (%)	53.4	47.1	53.0	56.9	54.3	44.7	.061
History of CAD (%)	16.7	20.6	14.7	17.5	12.2	23.5	.028
History of stroke (%)	15.9	13.2	16.3	16.6	15.9	15.2	.909
History and comorbidity of cancer (%)	10.6	8.8	12.1	10.3	6.5	15.9	.045
Dialysis							
Hemodialysis (%)^#^	92.9	94.9	90.7	93.1	93.9	94.7	.338
Arteriovenous fistula (%)^#^	82.6	84.4	81.4	83.2	84.3	78.8	.420
Arteriovenous graft (%)^#^	10.2	10.4	9.7	9.7	9.5	15.7	
Peritoneal access (%)^#^	7.2	5.2	9.0	7.1	6.2	5.5	
Medications							
Use of ACEIs/ARBs (%)	60.4	59.3	64.2	60.8	61.1	47.0	.013
Use of *β* blockers	34.7	31.6	29.1	37.8	35.9	41.7	.019
Use of statin (%)	40.1	39.0	42.1	41.4	39.2	31.1	.218
Use of allopurinol (%)	42.5	73.5	60.2	34.3	22.0	24.2	<.001
Use of loop diuretics (%)	66.2	64.7	65.6	67.0	63.3	71.2	.592
Use of thiazide (%)	22.9	15.4	17.9	23.0	29.0	34.8	<.001
Use of ESA (%)	86.9	88.2	90.4	86.0	86.9	78.0	.006
Laboratory data							
Hemoglobin (g/dL)	9.4, 1.8	9.3, 1.6	9.5, 1.4	9.4, 1.5	9.3, 1.6	9.4, 1.8	.352
Serum albumin (g/dL)	3.21, 0.59	3.12, 0.63	3.28, 0.60	3.20, 0.58	3.11, 0.59	3.28, 0.57	.002
Serum uric acid (mg/dL)	8.8, 2.4	5.0, 0.8	7.1, 0.5	8.9, 0.6	10.8, 0.5	14.1, 1.9	<.001
BUN (mg/dL)	91.7, 30.1	85.0, 33.1	87.1, 24.5	89.1, 26.5	97.4, 33.3	114.0, 38.7	<.001
Serum creatinine (mg/dL)	8.96, 3.14	8.26, 3.09	8.85, 2.74	8.91, 2.89	9.39, 3.44	9.49, 4.44	.003
eGFR (mL/min/1.73m^2^]	5.4, 2.2	6.0, 3.3	5.4, 2.0	5.4, 2.1	5.2, 2.0	5.4, 2.2	.019
Serum adjusted calcium (mg/dL)	8.6, 1.1	8.7, 1.0	8.6, 1.1	8.6, 1.1	8.6, 1.1	8.6, 1.1	.969
Serum phosphate (mg/dL)	6.4, 1.9	6.0, 1.8	6.1, 1.6	6.3, 1.7	6.7, 2.0	7.4, 2.4	<.001
Serum total cholesterol (mg/dL)	162, 44	157, 40	161, 41	162, 44	164, 46	169, 55	.236
CRP (mg/dL)*	0.30 [0.10 − 1.31]	0.20 [0.08 − 0.80]	0.35 [0.11 − 2.39]	1.28 [0.10 − 1.38]	0.30 [0.10 − 1.09]	0.76 [0.20 − 2.47]	.001
HCO_3_^–^ (mmol/L)	19.7, 4.9	19.0, 4.4	19.2, 4.3	19.8, 4.8	19.9, 5.2	20.8, 6.1	.017

Mean, standard deviation or %, *median [interquartile].

^#^Status at maintenance dialysis.

G1-5 was classified by serum uric acid level.

G1; 6.0 mg/dL <, G2; 6.0 <, < 8 mg/dL, G3; 8.0 <, < 10.0 mg/dL, G4; 10.0 <, < 12.0 mg/dL, G5; < 12.0 mg/dL.

BMI: body mass index; CAD: coronary artery disease; ACEI: angiotensin converting enzyme inhibitor; ARB: angiotensin receptor blocker; ESA: erythropoiesis stimulating agent; BUN: blood urea nitrogen; CRP: C-reactive protein

### Comparison of all-cause, infection-related, CV-related, and cancer-related mortality among the five groups

**Figure 1.**
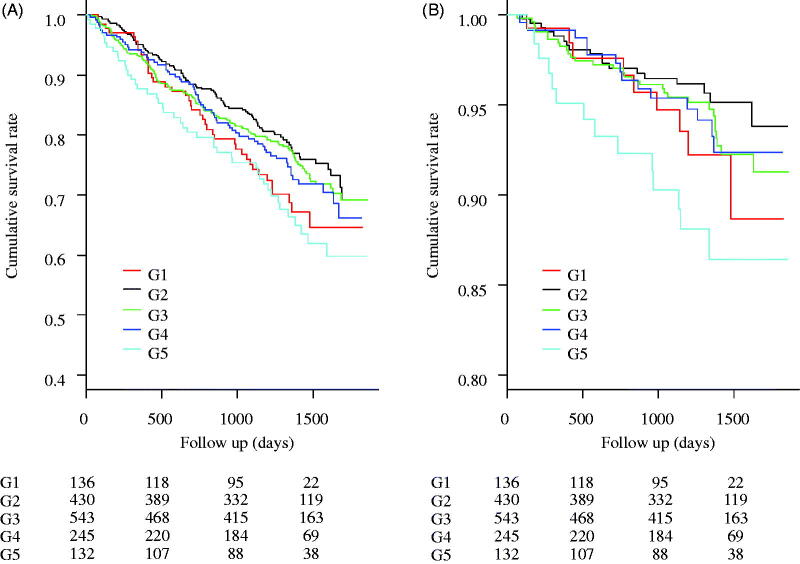
Kaplan–Meier curves for the cumulative survival rates between the 5 groups. (A) All-cause mortality. No significant differences were observed between the 5 groups’ cumulative survival rates (the Logrank test, *p* = .056). (B) Infection-related mortality. No significant differences were observed between the 5 groups’ cumulative survival rates (*p* = .064).

**Figure 2.**
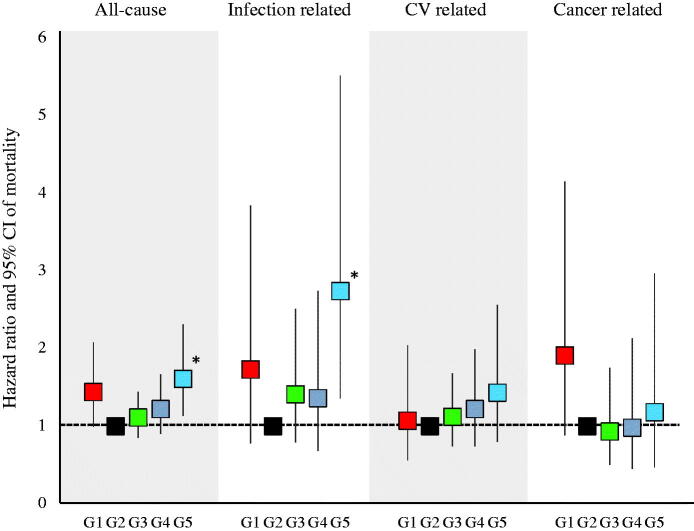
Hazard ratios for all-cause, infection related, CV related, and cancer-related mortality. All-cause and infection-related mortality were significantly higher in the G5.

There were 381 cases (25.6%) of all-cause deaths, 87 cases (5.9%) of infection-related deaths, 149 cases (10.0%) of CV-related deaths, and 65 cases (4.4%) of cancer-related deaths. Figure 1 shows the results of the comparison of all-cause and infection-related mortality using the log-rank test. No significant differences were observed in all-cause and infection-related mortality among the five groups (*p* = .056 and .064, respectively). Figure 2 shows the HRs for all-cause, infection-related, CV-related, and cancer-related mortality. All HRs were calculated relative to G2. All-cause and infection-related mortality were significantly higher in G5 (HR: 1.61, 95% confidence interval [CI]: 1.13–2.31 and HR: 2.74, 95% CI: 1.36–5.52, respectively) than in the other groups.

### Univariate analysis of factors affecting all-cause and infection-related mortality

Table 2 shows the HRs for all-cause and infection-related mortality as identified using the univariate regression analysis. Some variables, including age, sex, BMI, primary kidney disease, history of CAD and stroke, history or comorbidity of cancer, dialysis modality, vascular access, use of ACEIs/ARBs, allopurinol, and loop diuretics, eGFR, and hemoglobin, serum albumin, BUN, serum creatinine, serum adjusted calcium, serum phosphate, serum total cholesterol, and CRP levels in addition to the G5 referred to the G2 were associated with all-cause mortality. Meanwhile, age, sex, BMI, primary kidney disease, diabetes, history of stroke, history or comorbidity of cancer, vascular access, use of ACEIs/ARBs, eGFR, and serum albumin, serum creatinine, serum adjusted calcium, and CRP levels in addition to the G5 referred to the G2 were associated with infection-related mortality.

**Table 2.** Hazard ratio for all-cause and infection-related mortality by univariate regression analysis.

	All-cause mortality			Infection-related mortality		
Variables	HR	95% CI	*p* Value	HR	95% CI	*p* Value
G1 (reference to G2)	1.44	0.99−2.08	.055	1.73	0.78−3.84	.181
G3 (reference to G2)	1.11	0.85−1.44	.450	1.41	0.79−2.51	.246
G4 (reference to G2)	1.22	0.90−1.67	.206	1.36	0.68−2.74	.386
G5 (reference to G2)	1.61	1.13−2.31	.009	2.74	1.36−5.52	.005
Age (/10 years old)	1.90	1.71−2.10	<.001	2.50	1.97−3.18	<.001
Female gender	0.66	0.52−0.83	<.001	0.58	0.35−0.96	.033
BMI (/kg/m^2^)	0.92	0.89−0.94	<.001	0.85	0.80−0.91	<.001
Nephrology care (/100 d)	0.99	0.98−1.00	.058	0.99	0.98−1.01	.513
DMN (reference to CGN)*	2.08	1.38−3.12	<.001	1.10	0.47−2.58	.830
HNS (reference to CGN)*	3.43	2.27−5.18	<.001	3.99	1.79−8.90	.001
Others (reference to CGN)*	2.27	1.44−3.56	<.001	2.50	1.04−5.99	.040
Diabetes	1.01	0.83−1.24	.921	1.95	1.26−3.02	.003
History of CAD	1.67	1.32−2.12	<.001	1.43	0.85−2.41	.173
History of stroke	1.64	1.29−2.08	<.001	1.93	1.19−3.13	.008
History and comorbidity of cancer	1.77	1.35−2.33	<.001	1.78	1.01−3.16	.048
Hemodialysis (reference to PD)	3.20	1.71−6.00	<.001	22.82	0.75−691.7	.072
AVG (reference to AVF)^#^	1.65	1.24−2.19	.001	1.85	1.06−3.22	.031
Use of ACEIs/ARBs	0.71	0.58−0.87	.001	0.64	0.42−0.97	.035
Use of *β* blockers	1.12	0.91−1.38	.291	0.76	0.48−1.20	.239
Use of statin	0.83	0.67−1.02	.072	0.77	0.49−1.19	.234
Use of allopurinol	1.34	1.10−1.64	.004	1.39	0.92−2.12	.122
Use of loop diuretics	1.33	1.06−1.66	.013	1.48	0.92−2.39	.105
Use of thiazide	0.97	0.76−1.24	.826	0.96	0.58−1.60	.879
Use of ESA	0.76	0.58−1.01	.058	1.48	0.68−3.21	.320
Hemoglobin (/g/dL)	0.93	0.87−0.99	.028	0.93	0.81−1.06	.286
Serum albumin (/g/dL)	0.67	0.57−0.79	<.001	0.54	0.39−0.76	<.001
BUN (/10 mg/dL)	1.04	1.00−1.07	.028	1.04	0.97−1.11	.262
Serum creatinine (/mg/dL)	0.85	0.82−0.89	<.001	0.80	0.73−0.88	<.001
eGFR (/mL/min/1.73 m^2^)	1.12	1.08−1.15	<.001	1.13	1.07−1.20	<.001
Serum adjusted calcium (/mg/dL)	1.33	1.20−1.47	<.001	1.26	1.03−1.55	.028
Serum phosphate (/mg/dL)	0.90	0.85−0.96	.001	0.94	0.83−1.06	.285
Serum total cholesterol (/10 mg/dL)	0.97	0.95−1.00	.019	0.97	0.92−1.03	.305
CRP (/mg/dL)	1.04	1.02−1.06	<.001	1.04	1.01−1.08	.008
HCO_3_^−^ (/mmol/L)	1.02	1.00−1.05	.061	0.99	0.94−1.04	.641

HR: hazard ratio; CI: confidence interval

G1-5 was classified by serum uric acid level.

G1; 6.0 mg/dL <, G2; 6.0 <, <8 mg/dL, G3; 8.0 <, <10.0 mg/dL, G4; 10.0 <, <12.0 mg/dL, G5; < 12.0 mg/dL.

* Primary kidney disease, ^#^status on maintenance dialysis.

BMI: body mass i7ndex; DMN: diabetic nephropathy; CGN: chronic glomerulonephritis; HNS: hypertensive nephrosclerosis; CAD: coronary artery disease; PD: peritoneal dialysis; AVF: arteriovenous fistula; AVG: arteriovenous graft; ACEI: angiotensin-converting enzyme inhibitor; ARB: angiotensin receptor blocker; ESA: erythropoiesis-stimulating agent; BUN: blood urea nitrogen; CRP: C-reactive protein

### HRs for all-cause, infection-related, CV-related, and cancer-related mortality calculated using the multivariate analysis

Table 3 shows the HRs for all-cause and infection-related mortality calculated using the multivariate regression analysis. For Models 1 and 2, the all-cause mortality rate was significantly higher in G5 than in G2 (HR: 1.63, 95% CI: 1.14–2.33 and HR: 1.78, 95% CI: 1.19–2.68, respectively). For Models 1, 2, and 3, the infection-related mortality rate was significantly higher in G5 than in G2 (HR: 2.75, 95% CI: 1.37–5.54, HR: 3.09, 95% CI: 1.45–6.59, and HR: 3.37, 95% CI: 1.24–9.15, respectively). For Model 3, the cancer-related mortality rate was significantly higher in G1 than in G2.

**Table 3.** Comparison of all-cause, infection related, CV-related, and cancer-related death.

	All N, 1486	G1 N, 136	G2 N, 430	G3 N, 543	G4 N, 245	G5 N, 132
All-cause death*	381, 25.6	40, 29.4	96, 22.3	134, 24.7	67, 27.3	44, 33.3
Model 1		1.26 (0.87 − 1.82)	Reference	1.18 (0.91 − 1.53)	1.35 (0.99 − 1.84)	1.63 (1.14 − 2.33)***
Model 2		1.07 (0.72 − 1.60)	Reference	1.27 (0.96 − 1.69)	1.63 (1.16 − 2.30)***	1.78 (1.19 − 2.68)***
Model 3		0.80 (0.50 − 1.27)	Reference	1.03 (0.66 − 1.60)	1.41 (0.84 − 2.35)	1.44 (0.81 − 2.57)
Infection-related death*	87, 5.9	9, 6.6	18, 4.2	32, 5.9	14, 5.7	14, 10.6
Model 1		1.49 (0.67 − 3.31)	Reference	1.54 (0.86 − 2.74)	1.57 (0.78 − 3.15)	2.75 (1.37 − 5.54)***
Model 2		1.09 (0.47 − 2.57)	Reference	1.65 (0.91 − 3.01)	1.80 (0.85 − 3.84)	3.09 (1.45 − 6.59)***
Model 3		0.74 (0.28 − 1.97)	Reference	1.48 (0.61 − 3.59)	1.83 (0.63 − 5.29)	3.37 (1.24 − 9.15) **
CV-related death*	149, 10.0	12, 8.8	39, 9.1	55, 10.1	27, 11.0	16, 12.1
Model 1		0.96 (0.50 − 1.83)	Reference	1.18 (0.78 − 1.77)	1.32 (0.81 − 2.16)	1.45 (0.81 − 2.60)
Model 2		0.86 (0.44 − 1.67)	Reference	1.13 (0.73 − 1.76)	1.53 (0.90 − 2.60)	1.27 (0.64 − 2.53)
Model 3		1.34 (0.61 − 2.99)	Reference	1.45 (0.66 − 3.18)	1.93 (0.80 − 4.64)	1.57 (0.57 − 4.31)
Cancer-related death*	65, 4.4	10, 7.4	18, 4.2	21, 3.9	10, 4.1	6, 4.5
Model 1		1.67 (0.77 − 3.61)	Reference	1.03 (0.55 − 1.94)	1.11 (0.51 − 2.41)	1.20 (0.47 − 3.01)
Model 2		1.73 (0.74 − 4.02)	Reference	1.33 (0.66 − 2.69)	1.61 (0.68 − 3.82)	1.57 (0.53 − 4.61)
Model 3		0.34 (0.13 − 0.90)**	Reference	0.44 (0.18 − 1.08)	1.01 (0.38 − 2.65)	0.56 (0.16 − 1.93)

*Value, %, HR (95% CI).

**p < .05 and***p < .01.

G1-5 was classified by serum uric acid level.

G1; 6.0 mg/dL <, G2; 6.0 <, < 8 mg/dL, G3; 8.0 <, < 10.0 mg/dL, G4; 10.0 <, < 12.0 mg/dL, G5; < 12.0 mg/dL.

Model 1; adjusted for age and gender, Model 2; adjusted for Model 1 + diabetes, coronary artery disease, stroke, vascular access, ACEI/ARB, allopurinol, loop diuretics, thiazide, hemoglobin, albumin, eGFR, and CRP, Model 3; stepwise regression adjusted for Model 2 + BMI, primary kidney disease, cancer, dialysis modality, β-blockers, statin, ESA, BUN, creatinine, adjusted calcium, phosphate, total cholesterol, and HCO_3_^−^.

HR: hazard ratio; CI: confidence interval; CV: cardiovascular; ACEI: angiotensin-converting enzyme inhibitor; ARB: angiotensin receptor blocker; eGFR: estimated glomerular filtration rate; CRP: C-reactive protein; BMI: body mass index; ESA: erythropoiesis-stimulating agent; BUN: blood urea nitrogen

### Adjusted HRs for all-cause and infection-related mortality in G5 and G2 across subgroups

Table 4 shows the adjusted all-cause and infection-related mortality in G5 and G2 across subgroups according to the medications affecting the serum UA levels. Among patients taking diuretics and allopurinol, the all-cause mortality rate was significantly higher in G5 than in G2. Meanwhile, the infection-related mortality rate was significantly higher in G5 than in G2 except for the patients who did not use allopurinol.

**Table 4.** Adjusted HRs (95% CI) for all-cause and infection-related mortality between the G5 and G2 across subgroup according to medication affecting serum uric acid.

	HR	95% CI	*p* Value
All-cause mortality			
Loop diuretics (+)	1.82	1.13−2.91	.013
Loop diuretics (−)	1.72	0.73−4.06	.217
Loop diuretics or Thiazide (+)	1.70	1.07−2.71	.025
Loop diuretics or Thiazide (−)	1.70	0.71−4.08	.233
Allopurinol (+)	2.17	1.08−4.33	.029
Allopurinol (−)	1.34	0.77−2.33	.294
Infection-related mortality			
Loop diuretics (+)	2.53	1.01−6.33	.048
Loop diuretics (−)	5.55	1.23−24.96	.026
Loop diuretics or Thiazide (+)	2.60	1.05−6.43	.040
Loop diuretics or Thiazide (−)	5.55	1.23−24.96	.026
Allopurinol (+)	6.49	2.27−18.58	<.001
Allopurinol (−)	1.71	0.56−5.20	.346

HR: hazard ratio; CI: confidence interval

G1 and 5 was classified by serum uric acid level.

G2; 6.0 <, < 8 mg/dL, G5; < 12.0 mg/dL.

Adjusted for age and gender, diabetes, CAD, stroke, vascular access, ACEIs/ARBs, allopurinol, loop diuretics, thiazide, hemoglobin, albumin, eGFR, and CRP.

CAD: coronary artery disease; ACEI: angiotensin-converting enzyme inhibitor; ARB: angiotensin receptor blocker; CRP: C-reactive protein

## Discussion

This study showed that the serum UA level at the initiation of dialysis was associated with infection-related mortality. We believe that this is the first study that investigated the relationship between the serum UA level and infection-related mortality, rather than the CV-related mortality. In addition, in this study, we set the baseline as the time of dialysis initiation, whereas the other studies were limited to the pre-dialysis or maintenance dialysis periods. Although vascular access infection is one of the most important causes of infection-related mortality in dialysis patients, there were no patients with tunneled dialysis catheter in this study.

We believe that there are two possible reasons explaining the relationship between the serum UA level and infection-related mortality. First, the function of neutrophils in patients with high serum UA levels might be worse than in those with low serum UA levels. Serum UA acts as one of the most important antioxidants against pro-oxidants, such as hydroxyl free radicals in the plasma [34]. Akbar et al. showed that UA was a useful marker in evaluating the severity of conditions in patients with sepsis, and high serum UA levels reflected strong oxidative stress [27]. Neutrophils generate reactive oxygen species (ROS) to sterilize some bacteria and fungi. We speculated that dialysis patients with extremely high serum UA levels are likely to be more severely affected by infectious diseases, because neutrophils would be unable to generate enough ROS. Second, vascular damage in patients with high serum UA levels progresses and leads to CV disease. Comorbid CV disease is one of the risk factors for infection [35,36]. UA is produced in all cells from xanthine catalyzed by xanthine oxidase; ROS are also produced simultaneously [37]. An increase in intracellular UA levels leads to pro-oxidant effects, including mitochondrial injury, activation of the renin–angiotensin system, induction of senescence and apoptosis, increase in phosphorylation of platelet-derived growth factor receptor, and ATP depletion [38]. The pathophysiological effects are thought to be associated with vascular damage, which causes metabolic syndrome and CV events clinically.

In some previous studies, all-cause and CV-related mortality were found to be higher in patients with low serum UA levels; in addition, the mortality rate in patients with high serum UA levels was relatively lower than that of patients with low serum UA levels [24,26,39,40,41]. The results of previous studies were different from our results. We suppose that the differences arose from the cutoff values of the serum UA. Kim et al. indicated that the all-cause mortality rate was significantly low in patients with serum UA levels of ≥8.5 mg/dL [26]. Beberashvili et al. showed that the all-cause mortality rate was significantly low in hemodialysis patients with serum UA levels of ≥6.2 mg/dL [42]. Meanwhile, in our study, we divided the patients into five groups according to the serum UA levels, and the cutoff value of the group with the highest serum UA level was ≥12.0 mg/dL. The groups with the second and third highest serum UA levels in our study were comparable to the groups with serum UA levels of 6.2 and ≥8.5 mg/dL, respectively, in the previous studies. The all-cause mortality of these groups in our study was not very high. Therefore, we considered that patients with extremely high serum UA levels would have a poor survival. It is possible that there are other factors, such as malnutrition, associated with the higher mortality in patients with hypouricemia. Several studies have shown that a low serum UA level was related to nutritional and inflammatory markers [41,42,43]. Similarly, in this study, we could elucidate that the all-cause mortality in patients with the lowest serum UA level was high but not significantly higher than those of the other groups. The low levels of serum albumin, total cholesterol, and creatinine in the group with the lowest serum UA level might support this speculation.

UA-lowering agents, such as allopurinol, are often used in patients with CKD, including those undergoing dialysis. However, we cannot administer a sufficient dose of allopurinol to CKD patients, especially those undergoing dialysis, because of its side effects. Therefore, the serum UA level increased at dialysis initiation despite the use of allopurinol; in addition, the use of loop diuretics and thiazide played a role in increasing the serum UA level. The stratified analysis revealed that infection-related mortality was significantly higher in those taking allopurinol in this study. We considered that the results show that the serum UA levels in some patients were too high; thus, lowering the UA levels within an appropriate concentration range is difficult, despite the use of allopurinol. In our study, we could only use allopurinol for patients enrolled from 2011 to 2013, although other agents, such as febuxostat and topiroxostat, which might have fewer side effects compared to allopurinol, have been used more recently. Hence, it might be easier to reduce the serum UA level using febuxostat or topiroxostat in patients with advanced CKD. However, the Cardiovascular Safety of Febuxostat and Allopurinol in Patients with Gout and Cardiovascular Morbidities (CARES) trial indicated that all-cause and CV mortality were higher with febuxostat than with allopurinol [44]. Therefore, we should be careful enough to use UA-lowering agents.

There were limitations to this study. First, we compared the outcomes that were only based on the serum UA levels at dialysis initiation. However, the timing of dialysis initiation did not vary greatly among patients, because it was decided by nephrologists certified by the Japanese Society of Nephrology. Therefore, we were able to evaluate the serum UA levels of patients with similar kidney functioning and identify those who were likely to have high or low serum UA levels. Second, the use of UA-lowering agents depended on each nephrologist. For example, we could not identify the reasons why patients with high serum UA levels did not receive treatment with drugs or whether they had side effects or not. Third, this study was conducted in an observational manner, and the patients’ characteristics and laboratory data at dialysis initiation varied to some extent. The direct relationship between the serum UA levels and infection-related mortality could not be identified despite the adjustment of several variables. Fourth, there were possibilities of unknown or unmeasured confounding factors affecting the outcome in the multivariate regression analysis, although we used clinical variables that were easily obtained in real-world clinical settings.

In conclusion, this study showed that the serum UA level at dialysis initiation was associated with infection-related mortality in incident dialysis patients without use of tunneled dialysis catheters. Recently, we have also used several kinds of UA-lowering agents in patients with advanced CKD. Further studies are needed to clarify the relationship in a randomized-controlled manner in the future.

## Ethics approval and consent to participate

The AICOPP was conducted following Ethical guidelines for Clinical Research by the Japanese Ministry of Health, Labor, and Welfare (created 30 July 2003; full revision 28 December 2004; full revision 31 July 2008) and the Helsinki Declaration (revised 2013) and was approved by the clinical research ethics committees at each AICOPP group facility (approval number: 20110823-3). The subjects were given oral and written explanations of the aim of the study and provided consent in writing. Trial registration is UMIN 7096. Registered 18 January 2012.
